# MKL1 inhibits cell cycle progression through p21 in podocytes

**DOI:** 10.1186/s12867-015-0029-5

**Published:** 2015-02-12

**Authors:** Shuang Yang, Lingjia Liu, Pengjuan Xu, Zhuo Yang

**Affiliations:** Medical School, Tianjin Key Laboratory of Tumor Microenvironment and Neurovascular Regulation, Nankai University, 94 Weijin Road, Tianjin, 300071 China; Tianjin University of Traditional Chinese Medicine, Tianjin, 300193 China

**Keywords:** Kidney development, Podocyte, Cell growth arrest, MKL1

## Abstract

**Background:**

The glomerular podocyte is a highly specialized cell type with the ability to ultrafilter blood and support glomerular capillary pressure. However, little is known about the genetic programs leading to this functionality or the final phenotype.

**Results:**

In the current study, we found that the expression of a myocardin/MKL family member, MKL1, was significantly upregulated during cell cycle arrest induced by a temperature switch in murine podocyte clone 5 (MPC5) cells. Further investigation demonstrated that overexpression of MKL1 led to inhibition of cell proliferation by decreasing the number of cells in S phase of the cell cycle. In contrast, MKL1 knockdown by RNA interference had the opposite effect, highlighting a potential role of MKL1 in blocking G1/S transition of the cell cycle in MPC5 cells. Additionally, using an RT^2^ Profiler PCR Array, p21 was identified as a direct target of MKL1. We further revealed that MKL1 activated p21 transcription by recruitment to the CArG element in its promoter, thus resulting in cell cycle arrest. In addition, the expression of MKL1 is positively correlated with that of p21 in podocytes in postnatal mouse kidney and significantly upregulated during the morphological switch of podocytes from proliferation to differentiation.

**Conclusions:**

Our observations demonstrate that MKL1 has physiological roles in the maturation and development of podocytes, and thus its misregulation might lead to glomerular and renal dysfunction.

**Electronic supplementary material:**

The online version of this article (doi:10.1186/s12867-015-0029-5) contains supplementary material, which is available to authorized users.

## Background

Podocytes, also called visceral glomerular epithelial cells, are terminally differentiated cells overlaying the outer region of the glomerular basement membrane of renal glomeruli. These cells have several key functions including the prevention of proteinuria, synthesis of basement membrane components, regulation of glomerular filtration, and counteraction of intraglomerular hydrostatic pressure [1]. Podocyte injury is typically associated with proteinuria and progressive glomerulosclerosis [2].

Podocytes are derived from epithelial cells originating in the metanephric mesenchyme, which develop into postmitotic terminally differentiated cells, and therefore have similarities to neurons [3,4]. During glomerulogenesis, podocytes proliferate until the S-shape body stage and exit the cell cycle at the capillary loop stage [5,6]. Podocytes then acquire their fully differentiated phenotype, a process that is not complete until 1 week after birth in the mouse. Mature podocytes tightly regulate and maintain their quiescent and differentiated phenotype, and therefore lost podocytes cannot be replaced by proliferation of neighboring undamaged cells. Indeed, studies have shown that the inability to proliferate contributes to glomerular scarring [7]. Thus, the mechanism responsible for the cell cycle arrest that occurs during nephrogenesis may also participate in maintenance of cell cycle quiescence in mature podocytes.

Megakaryoblastic leukemia 1 (MKL1) was originally found in a study of a chromosomal translocation, t (1;22), which is closely related to the incidence of acute megakaryoblastic leukemia in infants and children [8,9]. MKL1 has been recently shown to be a member of a three-protein family that includes MKL2 and myocardin. These myocardin/MKL proteins serve as serum response factor (SRF) coactivators by binding to SRF and strongly activating SRF target genes [10-13]. In contrast to myocardin, which has cardiac and smooth muscle-specific expression [10,14-16], MKL1 and MKL2 are expressed in a wide range of embryonic and adult tissues [8,11,17-20]. MKL1 regulates many processes including muscle cell differentiation [17], cardiovascular development [18], remodeling of neuronal networks in the developing and adult brain [19], megakaryocytic differentiation and migration [21,22], modulation of cellular motile functions, and epithelial-mesenchymal transition [23-25]. Notably, there is increasing evidence of the involvement of the myocardin/MKL family in suppression of cell proliferation and cell cycle progression. Both myocardin and MKL1 exert anti-proliferative effects in various cell lines [26-28]. Therefore, unraveling the functional pathways in which these proteins have a role and furthering our comprehension of the cellular mechanisms intrinsic to their regulation of cell proliferation will become increasingly important.

In the present study, we found that MKL1 expression was upregulated during temperature-switched growth arrest in murine podocyte clone 5 (MPC5) cells. Overexpression of MKL1 resulted in inhibition of G1/S cell cycle progression in cell viability and EdU cell proliferation assays, whereas MKL1 knockdown had the opposite effect. We further demonstrated that MKL1 induced expression of the cyclin-dependent kinase inhibitor (CKI) p21 during the regulation of cell cycle arrest. Importantly, MKL1 expression was observed in podocytes of the mouse kidney during postnatal development, which was upregulated during the morphological switch of podocytes from proliferation to differentiation.

## Results

### Expression of MKL1 is upregulated during temperature-switched cell growth arrest in MPC5 cells

**Figure 1:**
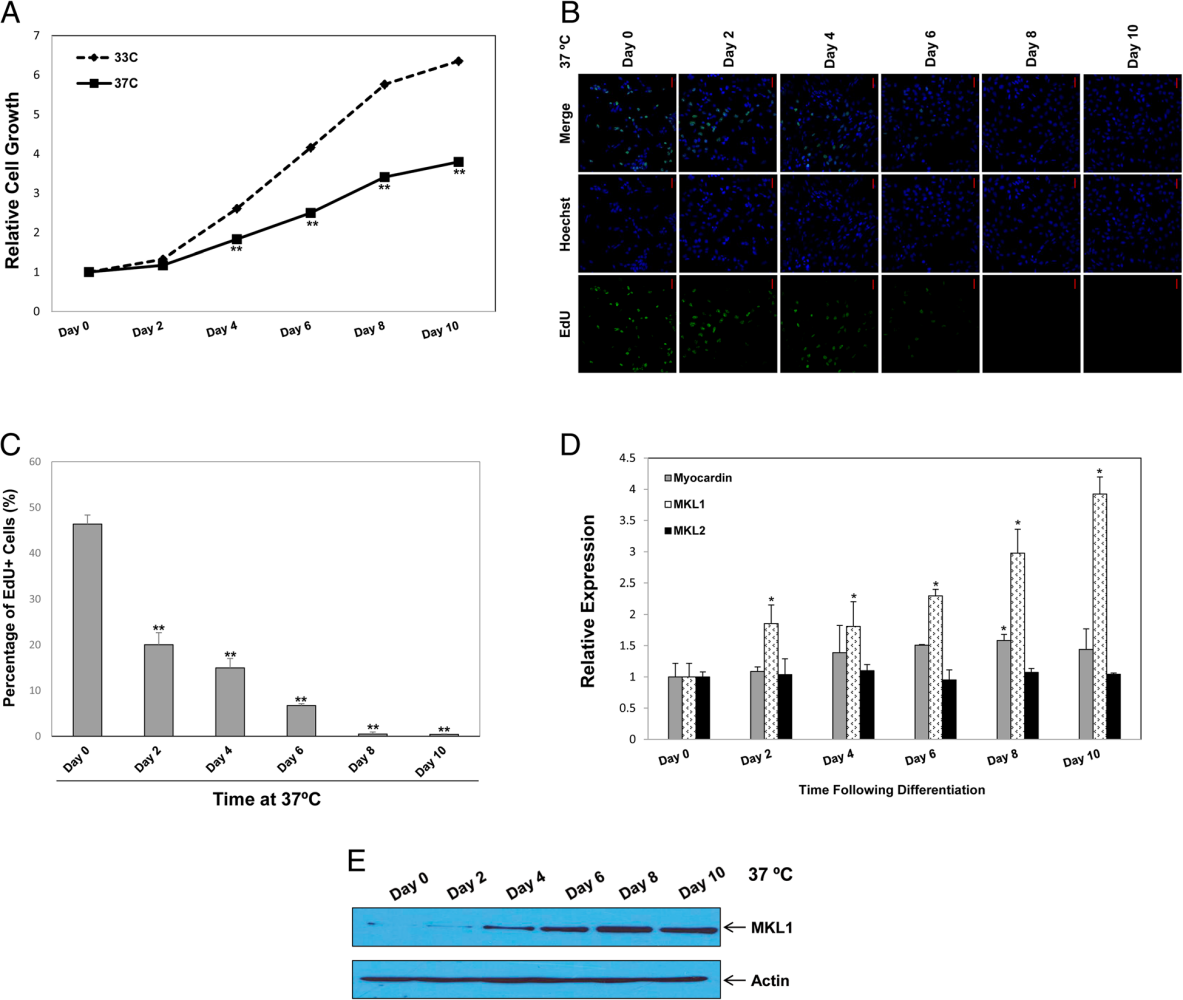
**MKL1 is upregulated during temperature-switched cell cycle arrest in MPC5 cells. A)** MPC5 cells were cultured at the permissive temperature of 33°C or the nonpermissive temperature of 37°C. At the indicated time points, cell growth was measured using a CCK-8 assay. **p < 0.01 compared with the control (one-way ANOVA followed by Tukey’s HSD test). **B)** MPC5 cells were shifted from 33°C to 37°C and cultured for the indicated times. Cell proliferation was measured by immunofluorescence analysis of EdU incorporation. Scale bars, 25 μm. **C)** The percentage of proliferating cells was calculated as EdU-positive cells/Hoechst-stained cells × 100%. **p < 0.01 compared with the control (one-way ANOVA followed by Tukey’s HSD test). **D)** MPC5 cells were shifted from 33°C to 37°C and cultured for the indicated times. The mRNA expression levels of myocardin, MKL1 and MKL2 were verified by qPCR. GAPDH was used to normalize expression levels. *p < 0.05 compared with the control (one-way ANOVA followed by Tukey’s HSD test). **E)** MPC5 cells were shifted from 33°C to 37°C and cultured for the indicated times. Protein expression levels of MKL1 were examined by western blotting. Actin was used to normalize MKL1 levels.

To assess the possible role of myocardin/MKL proteins in podocyte growth arrest, MPC5 cells were respectively maintained at the permissive temperature of 33°C and at the nonpermissive temperature of 37°C for 10 days. As shown in Figure 1A, a cell viability assay indicated that MPC5 cells cultured at 37°C showed a significant decrease in cell number compared with those cultured at 33°C. The results of immunofluorescence staining in the EdU cell proliferation assay further revealed a marked decrease in the number of cells in S phase by the temperature switch to 37°C (Figure 1B and C). The percentage of cells in S phase decreased from 46.37% to 20.03% as early as 2 days after the temperature switch. At 4–6 days, the percentage of cells in S phase further decreased from 14.97% to 6.70%, which is consistent with a previous report indicating that the temperature switch induces growth arrest of podocytes *in vitro* [29].

The expression of myocardin/MKL proteins was then measured during temperature-switched growth arrest in MPC5 cells. qPCR analysis indicated that the temperature switch to 37°C induced an approximate 1.8-fold increase in MKL1 mRNA expression compared with the basal level at 2 days (Figure 1D). At 4–10 days, MKL1 expression showed a 2-4-fold increase at the mRNA level. Western blotting was used to confirm the upregulation of MKL1 expression at the protein level (Figure 1E). However, the alteration in myocardin and MKL2 expression was not as evident (Figure 1D). Considering the dominant presence of MKL1 over its other family members, we focused on the effects of MKL1 in subsequent experiments.

### MKL1 functions as an effective inducer of cell growth arrest in MPC5 cells

**Figure 2:**
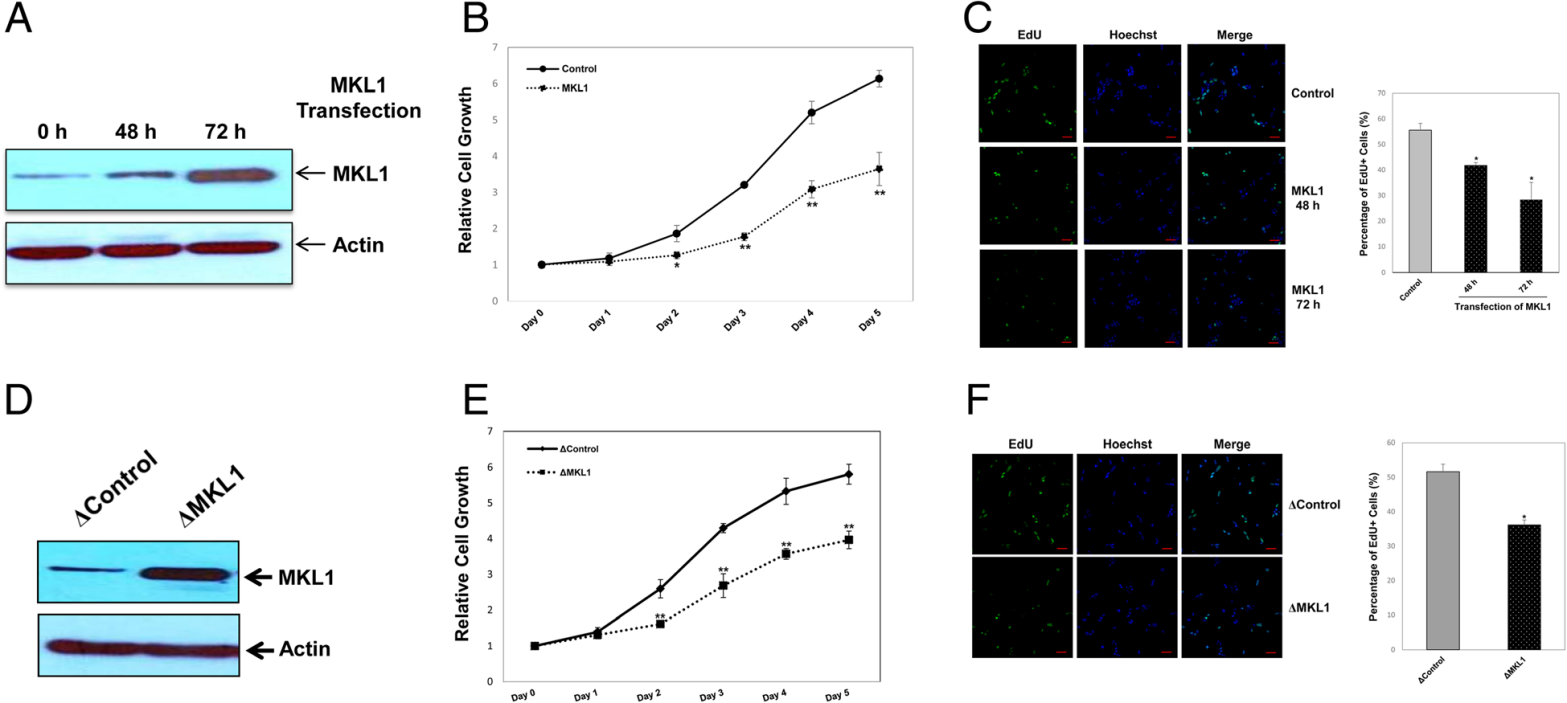
**Overexpression of MKL1 induces MPC5 cell growth arrest. A)** MPC5 cells were transiently transfected with a mouse MKL1 expression plasmid and cultured at 33°C. Expression of MKL1 protein was verified by western blotting. Actin was used to normalize MKL1 levels. **B)** At the indicated time points, cell growth was measured using the CCK-8 assay. *p < 0.05 and **p < 0.01 compared with the control (one-way ANOVA followed by Tukey’s HSD test). **C)** Cell proliferation was measured by immunofluorescence analysis of EdU incorporation. Scale bars, 25 μm. The percentage of proliferating cells was calculated as EdU-positive cells/Hoechst-stained cells × 100%. *p < 0.05 compared with the control (one-way ANOVA followed by Tukey’s HSD test). **D)** MPC5 cells were stably transfected with a mouse MKL1 expression plasmid and cultured at 33°C. Expression of MKL1 protein was verified by western blotting. Actin was used to normalize MKL1 levels. **E)** At the indicated time points, cell growth was measured using the CCK-8 assay. **p < 0.01 compared with the control (one-way ANOVA followed by Tukey’s HSD test). **F)** Cell proliferation was measured by immunofluorescence analysis of EdU incorporation. Scale bars, 25 μm. The percentage of proliferating cells was calculated as EdU-positive cells/Hoechst-stained cells × 100%. *p < 0.05 compared with the control (one-way ANOVA followed by Tukey’s HSD test).

Next, a mouse MKL1 expression plasmid [11] was transiently transfected into MPC5 cells. Overexpression of MKL1 was assessed by western blotting (Figure 2A). Compared with control cells, the cell viability assay indicated that ectopic expression of MKL1 inhibited MPC5 cell proliferation (Figure 2B). Results of DNA analysis by flow cytometry further confirmed that MKL1-overexpressing MPC5 cells had a lower population of S phase cells and a higher population of G0/G1 phase cells (Additional file 1: Figure S1). The EdU cell proliferation assay revealed a marked decrease in the number of S phase cells after MKL1 overexpression (Figure 2C). The percentage of cells in S phase decreased from 55.56% to 28.39% at 72 h after transfection of the MKL1 expression plasmid. Furthermore, MPC5 cells were stably transfected with either the MKL1 expression plasmid (ΔMKL1) or the empty vector (ΔControl). Overexpression of MKL1 was then examined by western blotting (Figure 2D). Cell viability and EdU cell proliferation assays confirmed that MKL1 overexpression induced a delay in G1/S phase transition of MPC5 cells (Figure 2E and F).

**Figure 3:**
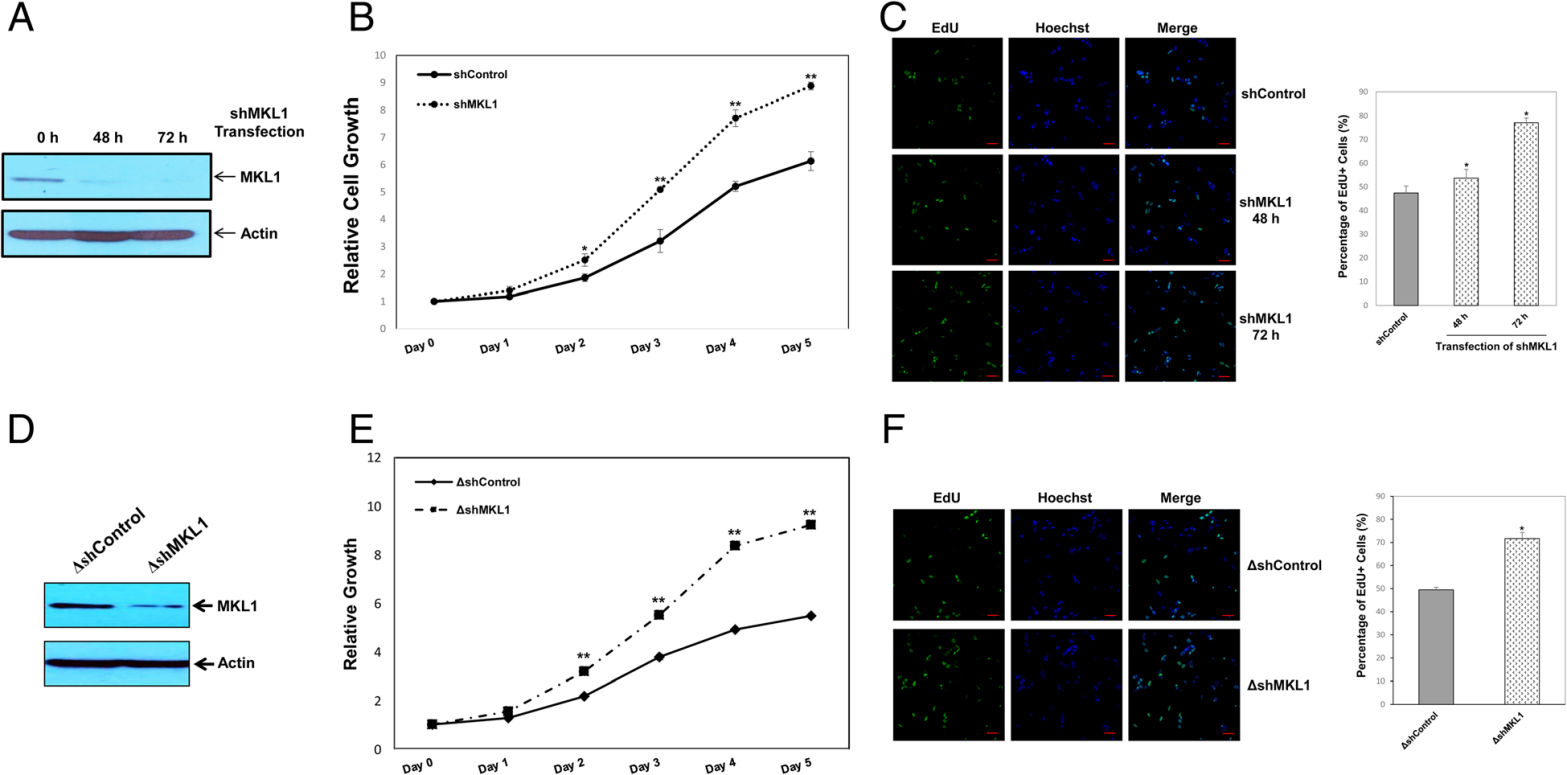
**Knockdown of MKL1 promotes cell cycle progression through S phase. A)** MPC5 cells were transiently transfected with a MKL1-specific shRNA plasmid (shMKL1) or a scrambled shRNA control plasmid (shControl) and cultured at 33°C. The efficiency of MKL1 protein knockdown was examined by western blotting. Actin was used to normalize MKL1 levels. **B)** At the indicated time points, cell growth was measured using the CCK-8 assay. * *p* < 0.05 and **p < 0.01 compared with the control (one-way ANOVA followed by Tukey’s HSD test). **C)** Cell proliferation was measured by immunofluorescence analysis of EdU incorporation. Scale bars, 25 μm. The percentage of proliferating cells was calculated as EdU-positive cells/Hoechst-stained cells × 100%. *p < 0.05 compared with the control (one-way ANOVA followed by Tukey’s HSD test). **D)** MPC5 cells were stably transfected with a MKL1-specific shRNA plasmid (ΔshMKL1) or a scrambled shRNA control plasmid (ΔshControl) and cultured at 33°C. Expression of MKL1 protein was verified by western blotting. Actin was used to normalize MKL1 levels. **E)** At the indicated time points, cell growth was measured using the CCK-8 assay. **p < 0.01 compared with the control (one-way ANOVA followed by Tukey’s HSD test). **F)** Cell proliferation was measured by immunofluorescence analysis of EdU incorporation. Scale bars, 25 μm. The percentage of proliferating cells was calculated as EdU-positive cells/Hoechst-stained cells × 100%. *p < 0.05 compared with the control (one-way ANOVA followed by Tukey’s HSD test).

Therefore, we hypothesized that knockdown of MKL1 by RNA interference would result in an increase in the number of cells in S phase. To test our hypothesis, a MKL1-targeting shRNA plasmid (shMKL1) or a scrambled control shRNA plasmid (shControl) were transiently transfected into MPC5 cells. Knockdown of MKL1 expression was confirmed by western blotting (Figure 3A). Compared with shControl cells, the cell viability assay indicated that depletion of MKL1 promoted MPC5 cell proliferation (Figure 3B). The results of DNA analysis by flow cytometry further showed that MKL1 knockdown MPC5 cells had a higher population of cells in S phase and a lower population of cells in G0/G1 phase compared with control cells (Additional file 2: Figure S2). The EdU cell proliferation assay revealed that repression of MKL1 resulted in a significant increase in the number of cells in S phase from 47.40% to 77.07% at 72 h after MKL1 knockdown (Figure 3C). In addition, MPC5 cells were stably transfected with either shMKL1 (ΔshMKL1) or shControl (ΔshControl). Knockdown of MKL1 expression was then confirmed by western blotting (Figure 3D). The cell viability and EdU cell proliferation assays confirmed that repression of MKL1 remarkably promoted cell cycle progression through S phase in MPC5 cells (Figure 3E and F).

### MKL1 regulates podocyte proliferation by targeting p21

**Figure 4:**
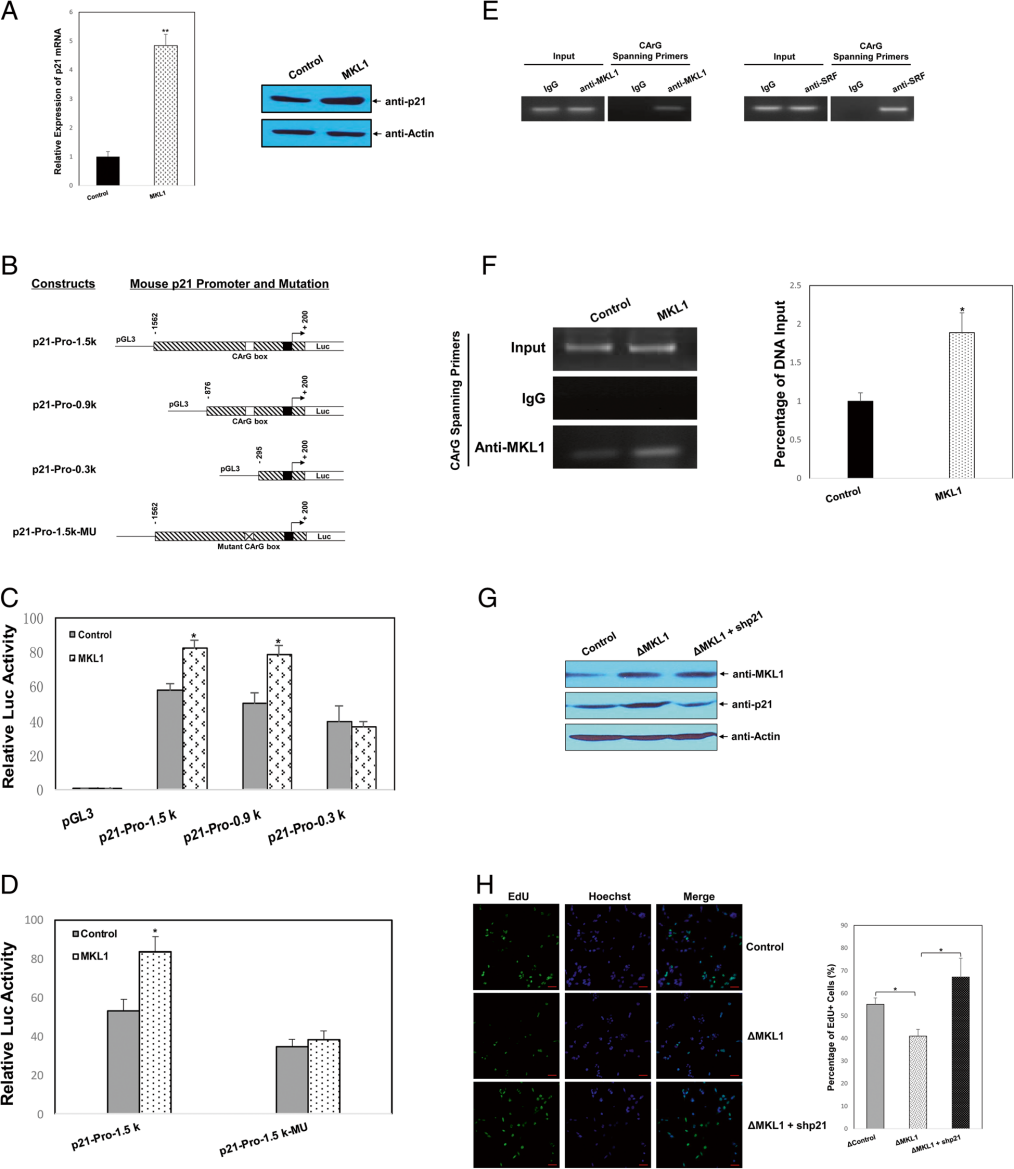
**MKL1 induces p21 expression. A)** The expression of p21 was examined by qPCR and western blotting in MKL1-overexpressing MPC5 cells. GAPDH and actin were used to normalize p21 levels. **p < 0.01 compared with the control (unpaired Student’s *t*-test). **B)** Sequential deletions and mutations of the mouse p21 promoter were fused to a luciferase reporter. MPC5 cells in 24-well plates were co-transfected with the MKL1 expression plasmid (1 μg/well) and various wild-type **C)** or mutant **D)** p21 promoter luciferase reporters (1 μg/well). The luciferase activity of the extracts was determined at 24 h after transfection using a Betascope analyzer. Luciferase values were normalized to Renilla activities. *p < 0.05 compared with the empty vector (unpaired Student’s *t*-test). **E)** ChIP assays were performed using an anti-MKL1 antibody, anti-SRF antibody, or control IgG in MPC5 cells. The association of MKL1 or SRF with the proximal mouse p21 promoter was analyzed by PCR. The amount of input was confirmed by equal loading of chromatin. **F)** ChIP assays were performed using the anti-MKL1 antibody or control IgG in MKL1-overexpressing MPC5 cells. The association of MKL1 with the proximal mouse p21 promoter was analyzed by PCR or qPCR. The amount of input DNA was confirmed by equal loading of chromatin. *p < 0.05 compared with the empty vector (unpaired Student’s *t*-test). **G)** ΔMKL1 cells were transiently transfected with a p21-specific shRNA plasmid (shp21) and cultured at 33°C. The efficiency of p21 knockdown was examined by western blotting. Actin was used to normalize MKL1 and p21 levels. **H)** Cell proliferation was measured by immunofluorescence analysis of EdU incorporation. Scale bars, 25 μm. The percentage of proliferating cells was calculated as EdU-positive cells/Hoechst-stained cells × 100%. *p < 0.05 compared with the control (one-way ANOVA followed by Tukey’s HSD test).

To identify the potential cellular pathways regulated by MKL1, differences in the mRNA levels of selected signaling molecules were examined using an RT^2^ Profiler PCR Array by comparing MKL1-expressing MPC5 cells with the control cells. We observed alterations in the expression of several cell cycle regulators, including p21, Gadd45a, Ddit3, E2F2, and cyclin A1 (Table 1). qPCR and western blotting were performed to verify these findings (Figure 4A and Additional file 3: Figure S3).

**Table 1:** **Genes regulated by MKL1**

**Unigene**	**GeneBank™ accession no.**	**Symbol**	**Description**	**Fold change (MKL1 vs. Control)**	***P*** **value**
Mm.195663	NM_007669	Cdkn1a	Cyclin-dependent kinase inhibitor 1A (p21)	4.8402	0.0317
Mm.72235	NM_007836	Gadd45a	Growth arrest and DNA-damage-inducible 45 alpha	2.9501	0.0268
Mm.110220	NM_007837	Ddit3	DNA-damage inducible transcript 3	2.5385	0.0282
Mm.307932	NM_177733	E2F2	E2F transcription factor 2	-8.3195	0.0146
Mm.4815	NM_007628	Ccna1	Cyclin A1	-6.1312	0.0195

Considering that MKL1 functions with its co-factor SRF by binding to the CArG box in the promoter region of target genes [12,13], we performed a search of the transcription factor database TRANSFAC and identified a CArG box (CCTTTTCTGG) at position −316/-307 in the mouse p21 promoter (Figure 4B). Thus, we assessed whether MKL1 was a bona fide activator of p21 transcription using reporter gene assays. As shown in Figure 4C, MKL1 significantly increased mouse p21 promoter activity of the wild-type −1562/+200 reporter by approximately 49% relative to the control without MKL1 transfection. Furthermore, we found that MKL1 activated the promoter activity of p21 in a dose-dependent manner (Additional file 4: Figure S4). A series of truncated p21 promoter-reporter constructs were thus generated for analysis, as shown in Figure 4B. The results showed that deletion of the CArG box significantly abolished MKL1-induced transactivation of the p21 promoter compared with that in the control without MKL1 transfection (Figure 4C). Next, we prepared mutants of the CArG box (CCTTTTCT*GG* to CCTTTTCT*TT*) by site-directed mutagenesis. We found that mutation of the CArG box was sufficient to interfere with MKL1-activated transcription of the p21 promoter (Figure 4D). Chromatin immunoprecipitation (ChIP) assays were then performed using an anti-MKL1 antibody, anti-SRF antibody, or control IgG in MPC5 cells. The results indicated that both MKL1 and SRF were able to bind to the p21 promoter during basal conditions in a CArG-dependent manner (Figure 4E). Overexpression of MKL1 resulted in a 1.9-fold increase in its binding to the endogenous p21 promoter in qChIP analysis (Figure 4F). These results suggested that the overexpressed MKL1 in conjunction with SRF promotes p21 transcription by binding to the CArG box in its promoter.

Importantly, to further show that MKL1 regulates podocyte proliferation by targeting p21, MKL1-overexpressing MPC5 cells (ΔMLK1) were transfected with a p21-targeting shRNA plasmid (shp21). Expression of MKL1 and p21 was assessed by western blotting (Figure 4G). The EdU cell proliferation assay revealed a marked decrease in the number of cells in S phase after MKL1 overexpression, whereas p21 interference remarkably attenuated the MKL1-inhibited cell cycle progression through S phase. The percentage of cells in S phase increased from 41.00% to 67.19% at 48 h after transfection of shp21 in MKL1-overexpressing MPC5 cells (Figure 4H). These observations confirmed that MKL1 inhibits MPC5 cell proliferation, which is effectively mediated by targeting p21.

### The expression of MKL1 and p21 is positively correlated in podocytes *in vivo*

**Figure 5:**
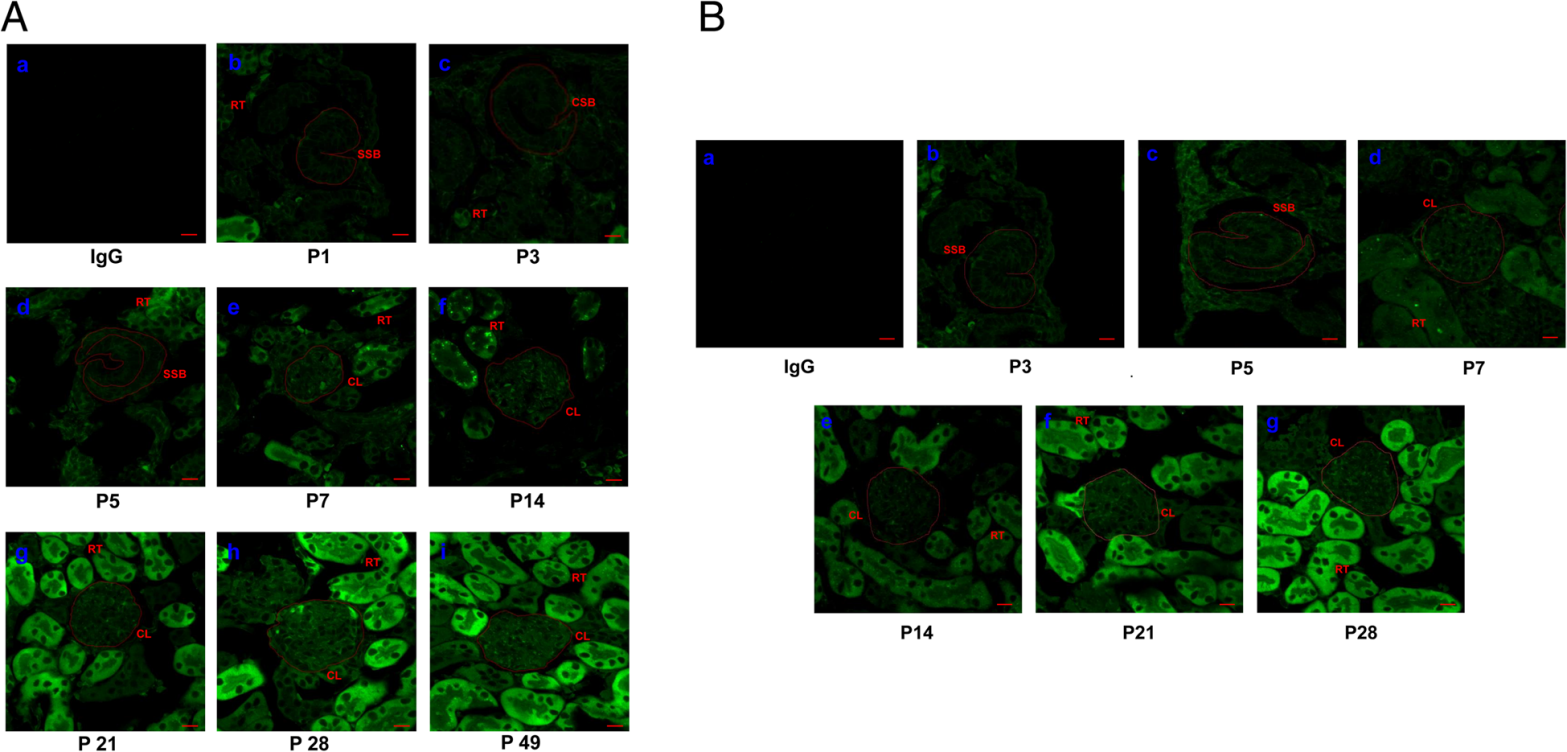
**The expression of MKL1 and p21 is correlated in podocytes** ***in vivo*** **. A)** MKL1 expression was examined by immunofluorescence staining of the mouse renal cortex at P1 (b), P3 (c), P5 (d), P7 (e), P14 (f), P21 (g), P28 (h), and P49 (i). The negative control image shows the renal cortex stained with a species-appropriate IgG (a). Scale bar, 50 μm. **B)** The expression of p21 was examined by immunofluorescent staining in the mouse renal cortex at P3 (b), P5 (c), P7 (d), P14 (e), P21 (f) and P28 (g). The negative control image was from the renal cortex, in which the primary antibody was a species-appropriate IgG (a). Scale bar, 50 μm. The S-shaped body (SSB), comma-shaped body (CSB), capillary loop (CL), and renal tubule (RT) are indicated.

Next, we detected the appearance of MKL1 in developing podocytes by examining its expression in the newborn mouse kidney that displays glomeruli at various stages of development from the S-shaped body through the capillary loop stage to mature glomeruli [5,6]. Immunofluorescence was used to detect MKL1 expression in the mouse kidney at postnatal day (P) 1, 3, 5, 7, 14, 21, 28 and 49. As seen in Figure 5A b-e, S-shaped and comma-shaped bodies were observed in the renal cortex of immature mice at P1–5, ultimately vascularizing into a capillary loop of mature nephron at P7. Moreover, MKL1 expression was found at all stages of renal glomerulus and tubule formation during postnatal development (Figure 5A b-i). Importantly, there was an increase in the expression of MKL1 in glomeruli between P5 and P7 (Figure 5A d-e).

Consequently, to address the correlation between MKL1 and p21 expression in newborn mouse kidney, immunofluorescent staining was used to detect p21 expression at P3-28. The results showed a relatively weak expression of p21 in glomeruli at P3-7 during the postnatal development (Figure 5B b-d). A remarkable upregulation of p21 expression was then observed between P14 and P21 (Figure 5B e-f), indicating a positive and sequential correlation between MKL1 and p21 expression. Considering that podocytes proliferate until the S-shape body stage and exit the cell cycle at the capillary loop stage during glomerulogenesis, these results are consistent with our notion that MKL1 functions as an effective inducer of cell growth arrest in podocytes, that might be mediated by the regulation of p21.

## Discussion

Growth arrest and differentiation of podocytes are essential for normal formation of glomeruli in the developing kidney and paramount for normal glomerular function in the mature kidney. The precise cell cycle regulation necessary to establish podocyte quiescence during development has not been fully defined. In the present study, we identified the contribution of one of the myocardin/MKL proteins, MKL1, to the regulation of MPC5 cell proliferation. During temperature-switched growth arrest of MPC5 cells, MKL1 expression was significantly upregulated above its other family members. Consequently, experiments were performed to assess gain- and loss-of-function of MKL1 to study the effect of MKL1 on MPC5 cell proliferation. We found that overexpression of MKL1 resulted in significant repression of G1/S phase progression of the cell cycle, whereas MKL1 knockdown had the opposite effect. Importantly, we demonstrated that MKL1 activated transcription of the cell cycle inhibitor p21 by recruitment to its promoter in a CArG element-dependent manner. In addition, MKL1 expression in the mouse kidney during postnatal development showed that MKL1 is expressed in podocytes *in vivo*, which was significantly upregulated during the morphological switch of podocytes from proliferation to differentiation.

Recent evidence supports an important and complex role of MKL1 in maintenance of proper differentiation in various cell lines including smooth muscle cells [30], myofibroblasts [31], megakaryocytes [22], and adipocytes [32]. Whether MKL1 functions in cell proliferation, however, remains poorly understood. Shaposhnikov *et al*. reported that MKL1 regulates the expression levels of two proapoptotic Bcl-1 family members, Bok and Noxa, and thus is involved in apoptotic signaling in NIH3T3 fibroblasts [33]. Here, we extended the study to show that elevated expression of MKL1 significantly blocked cell cycle progression by decreasing the number of MPC5 cells in S phase. On the other hand, RNA interference of MKL1 expression resulted in the opposite effect to promote G1/S transition of the cell cycle, confirming a potential role of MKL1 in regulation of cell proliferation. These observations collectively position MKL1 as a potential regulatory factor at the crossroad between cell proliferation and differentiation.

Indeed, myocardin family members have been previously implicated in the regulation of cell proliferation. Myocardin impairs the proliferation of vascular smooth muscle cells, Chinese hamster ovarian (CHO) cells, and leiomyosarcoma cells [27,28]. In HT1080 fibrosarcoma cells, myocardin inhibits proliferation at a low cell density and abrogates colony formation in soft agar [34]. However, the suggested molecular mechanisms underlying myocardin/MKL-induced cell growth arrest are conflicting. In human uterine leiomyosarcoma cells, myocardin in conjunction with SRF directly binds to the p21 promoter and induces its expression, thus resulting in G1/S arrest [27]. In contrast, myocardin has been shown to downregulate expression of c-myc, CDK1, CDK2, and S6K, but not p21 and p27, which leads to G2/M arrest and accumulation of polyploidy cells [35]. Our data presented here support the notion that MKL1 functions as an effective regulator to inhibit cell proliferation by altering p21 expression in MPC5 cells. Significant upregulation of p21 expression at both mRNA and protein levels was observed after transfecting the MKL1 expression plasmid, whereas MKL1 interference resulted in the opposite effect to downregulate p21 expression (Additional file 5: Figure S5), suggesting that a direct mechanism is involved in this regulation. We further demonstrated that MKL1 induced promoter activity of the *p21* gene in a dose-dependent manner. Importantly, we found that deletion or mutation of the CArG element in the mouse p21 promoter remarkably abolished the stimulatory effect on p21 transcription induced by MKL1. Transfection of the MKL1 expression plasmid led to a marked increase in the binding affinity of MKL1 for the endogenous p21 promoter, indicating a significant role of the CArG element in mediating MKL1-induced expression of p21. In addition to p21, we identified obvious candidates involved in MKL1-regulated MPC5 cell proliferation, such as Gadd45a, Ddit3, E2F2, and cyclin A1. However, these genes are not potential targets of myocardin/MKLs/SRF (unpublished data). These results indicate that an SRF-independent mechanism might contribute to MKL-mediated G1/S arrest of the cell cycle.

In the present study, we found that MKL1 was expressed in podocytes of the mouse kidney during postnatal development. Moreover, a significant increase in MKL1 expression was observed between P5 and P7 during postnatal development of the kidney, highlighting a potential role of MKL1 in the physiological and morphological switch of podocytes from proliferation to differentiation. Therefore, using the conditionally immortalized mouse podocyte cell line MPC5, we further revealed that MKL1 functioned as an effective inducer to inhibit cell proliferation and trigger cell cycle arrest at G1/S transition. Several studies have also demonstrated the presence of an intrinsic barrier to replication associated with activation of the cell cycle in podocytes. Re-expression of cell cycle proteins has been reported during glomerular disorders. *De novo* cyclin A staining is observed in podocytes of children with collapsing glomerulopathy [36] and focal segmental glomerulosclerosis (FSGS) [37]. Positive signals for cyclin D have also been reported in the cellular lesions of FSGS [38]. Recently, strong upregulation of CKIs p21 and p27 was reported in podocytes during Heymann nephritis and in diabetic ZDF-fa/fa rats [39,40]. Moreover, the glomerular tufts in crescentic glomerulonephritis strongly express CKIs [41], suggesting that podocytes upregulate CKIs to maintain cell cycle quiescence and preserve normal physiological functions. Here, we extended the study showing that MKL1 acted as an upstream regulator of a variety of cell cycle factors, such as p21 and cyclin A1, to control cell cycle progression in podocytes. In addition, we found significant upregulation of MKL1 expression in the renal tubular cells of newborn mouse kidneys. Recent reports have revealed a potential role of MKL1 in the regulation of renal tubular diseases. For example, Xu *et al.* reported that MKL1 is induced by glucose and synergizes with glucose to induce collagen expression in cultured renal tubular epithelial cells and the kidneys of mice with diabetic nephropathy, eventually leading to tubulointerstitial fibrosis [42]. Moreover, suppressor of cancer cell invasion (SCAI) has been demonstrated to negatively regulate epithelial-mesenchymal transition and renal fibrosis in LLC-PK1 (CL4) proximal tubular epithelial cells, which is at least partially mediated by repression of MKL1 and MKL2 [43]. Taken together, these observations indicate that MKL1 performs physiological roles in maturation and development of the kidney, and thus its dysfunction might lead to glomerular and renal diseases.

## Conclusion

In the present study, we found a potential mechanism of MKL1/p21-mediated cell cycle quiescence in podocytes. Therefore, these findings reveal a novel function of MKL1 during podocyte proliferation and differentiation, furthering our understanding of kidney development and the mechanisms of kidney diseases.

## Methods

### Plasmid construction

The mouse p21 promoter sequence (−1562/+200) was obtained by PCR from mouse blood genomic DNA and cloned into the pGL3-basic vector (Promega) using the forward primer, 5′-AGCAAGAATTCACAGACCGATG-3′, and reverse primer, 5′-GTACCTGACACATACACACC-3′. The mutagenesis of the CArG box in the mouse p21 promoter was performed using the QuickChange Site-Directed Mutagenesis Kit (Stratagene) with the forward primer: 5′-gtactcccctgtCCTTTTCT*TT*gaagtggtgatt-3′ and reverse primer: 5′-aatcaccacttc*AA*AGAAAAGGacaggggagtac-3′.

### Cell culture and transfection

MPC-5 cells were propagated in collagen I-coated dishes at 33°C (permissive temperature) in RPMI supplemented with 10% FBS and 20 U/ml of recombinant mouse IFN-γ (R&D). To induce differentiation, the medium was changed to RPMI with 5% FBS without IFN-γ, and the cells were shifted to 37°C (nonpermissive temperature) for 10 days. Under these conditions, cells underwent growth arrest, increased in size, and developed elongated cell processes. The cells were transfected using TurboFect™ Transfection Reagent (Fermentas) according to the manufacturer’s protocols.

The mouse MKL1 expression plasmid was introduced into MPC5 by transient transfection. G418-resistant clones were isolated over a period of 3–4 weeks. The overexpression of MKL1 was confirmed by western blotting assay.

### Cell viability assay

MPC5 cells transfected with the mouse MKL1 expression plasmid or empty vector were seeded onto 96-well plates at a density of 2 × 10^3^ cells/well and incubated in RPMI containing 5% FBS at 33°C for 6 days. The cell viability was assessed using the CCK-8 assay according to the manufacturer’s protocols (Dojindo). Six parallel replicates were measured for each sample.

### 5-ethynyl-2′-deoxyuridine (EdU) cell proliferation assay

MPC5 cells transfected with the mouse MKL1 expression plasmid or empty vector were seeded onto 24-well plates at 50-60% confluence. Cells were incubated with 50 μM EdU for 2 h at 48–72 h after transfection. After the 2-h pulse, the cells were washed twice with PBS and fixed with 4% paraformaldehyde at room temperature for 30 min. The cells were subsequently washed with glycine (2 mg/ml) for 5 min, added 0.2% Trion X-100 for 10 min, washed with PBS twice, and added click reaction buffer (Tris–HCl, pH 8.5, 100 mM; CuSO4, 1 mM; Apollo 550 fluorescent azide, 100 mM; ascorbic acid, 100 mM) for 30 min while protecting from light. The cells were then washed again with 0.5% Triton X-100 for three times, stained with Hoechst (5 mg/ml) for 30 min at room temperature, washed with 0.5% Triton X-100 for three times. Images were taken and analyzed using Confocal FV1000 (Olympus). EdU positive cells were calculated with (EdU add-in cells/Hoechst stained cells) × 100%. At least 200 cells were counted per well.

### RNA extraction and quantitative RT-PCR

Using TRIzol Reagent (Invitrogen), total RNA was extracted from MPC5 cells that were cultured at the nonpermissive temperature of 37°C for 10 days. Total RNA (0.5 μg) from each sample was used for first-strand cDNA synthesis using M-MLV Reverse Transcriptase (Promega). The specific products of mouse myocardin, MKL1 and MKL2 were amplified by quantitative PCR using the following primers: myocardin, 5′-GATGGGCTCTCTCCAGATCAG-3′ (forward) and 5′-GGCTGCATCATTCTTGTCACTT-3′ (reverse); MKL1, 5′-CCCAAAGGTAGCAGACAGTTC-3′ (forward) and 5′-GAGTGGGTGATATGGAGGTGG-3′ (reverse); and MKL2, 5′-GAGCGAGCCAGAACTGAGAAT-3′ (forward) and 5′-ACTCGAATCCACAGGAAGGATG-3′ (reverse). The verification of gene expression levels was performed by quantitative RT-PCR using EvaGreen (Biotium). GAPDH was used as an internal control.

### Western blotting and antibodies

Whole cell extract preparation and western blotting with the appropriate antibodies were performed as previously described [44]. The following antibodies (Abs) were used: rabbit polyclonal Ab against MKL1 at 1:800 dilution (ab49311; Abcam), rabbit polyclonal Ab against p21 at 1:500 dilution (10355-1-AP; Proteintech) and mouse monoclonal Ab against actin at 1:1000 dilution (A-4700; Sigma).

### RT^2^ Profiler™ PCR array

Total RNA was extracted from MPC5 cells stably transfected with the mouse MKL1 expression plasmid (ΔMKL1) or empty vector (ΔControl). For PCR array experiments, an RT^2^ Profiler custom PCR array was used to simultaneously examine the mRNA levels of 84 genes closely associated with cell cycle, including 5 “housekeeping genes”, in 96-well plates following the manufacturer’s protocol (PAMM-020Z, QIAGEN). Briefly, first-strand cDNAs were synthesized from 1 μg of total RNA using the TaqMan RT reagent kit (QIAGEN) according to the manufacturer’s protocol. The reaction mixtures (25 μl) were incubated at 25°C for 10 min, followed by incubation at 48°C for 30 min and 95°C for 5 min, then cooled on ice. Arrays were performed independently at least three times for each cell line; values were obtained for the threshold cycle (Ct) for each gene and normalized using the average of five housekeeping genes on the same array (Actb, B2m, Gapdh, Gusb, and Hsp90ab1). The Ct values for housekeeping genes and a dilution series of ACTB were monitored for consistency between the arrays. The change (ΔCt) between ΔMKL1 and ΔControl was determined using the formula ΔCt = Ct(ΔMKL1) – Ct (ΔControl), and the fold change was determined using the formula fold change = 2^(−ΔCt)^. The resulting values were reported as fold change; only genes showing twofold or greater change were considered. The negative controls ensured the absence of DNA contamination and set the threshold for determining absence versus presence of expression.

### Luciferase assay

MPC5 cells were co-transfected with the wild-type or mutant mouse p21 promoters and MKL1 expression plasmid in 24-well plates. Lysates were prepared at 24 h after transfection, and luciferase activities were measured using the Dual-Luciferase Reporter Assay System (Promega) according to the manufacturer’s protocols. The luciferase activities were normalized to the values for Renilla luciferase.

### Chromatin immunoprecipitation (CHIP)

ChIP assays were performed using reagents commercially obtained from Upstate, essentially according to the manufacturer’s instructions. The antibodies used in these experiments were rabbit polyclonal Ab against MKL1 (ab49311; Abcam) and anti-rabbit normal IgG (sc-2345, Santa Cruz). The amounts of each specific DNA fragment in immunoprecipitates were determined by PCR or quantitative PCR reactions. The fragment of mouse p21 promoter, containing the CArG box, was amplified using the forward primer: 5′-CCCTCGTGCTTAGACCA-3′, and reverse primer: 5′-GCTGTTGCTGCTACCCA-3′.

### Immunofluorescent analysis

Tissue samples were placed in 4% paraformaldehyde in PBS over 2 hours at 4°C, immersed in 30% sucrose overnight at 4°C, and then were embedded in OCT compound (Tissue-Tek, Miles) and sectioned at 5 mm (Leica CM 1850, Leica Instruments) for the morphological and immunohistochemistry study. Sections were washed three times in PBS for five minutes each and incubated in blocking buffer with 10% serum of the secondary antibody host species for 1 hour at room temperature. After that, sections were incubated with rabbit polyclonal Ab against MKL1 (ab49311; Abcam) and p21 (10355-1-AP; Proteintech) at 1:100 dilution overnight at 4°C. After being washed three times for 10 minutes with PBS at room temperature, sections were incubated with Alexa 488-conjugated anti-rabbit IgG for 3 hours at room temperature. Negative control samples were treated with species-appropriate IgG instead of primary antibody. Images were taken and analyzed using Confocal FV1000 (Olympus).

### Statistical analysis

SPSS 17.0 software (SPSS) was used for statistical analysis. The data from all the experiments are presented as means ± SD and represent three independent experiments. One-way analysis of variance (ANOVA) was used to compare means between treatment groups and Tukey’s HSD (honestly significant difference) test was used to evaluate the statistically significant differences between groups. Where appropriate, Student’s *t*-test for unpaired observations was applied. A *p*-value < 0.05 was considered significant.

## Additional files

Additional file 1: Figure S1. Overexpression of MKL1 induces cell cycle delay at the G1-S phase transition. Additional file 2: Figure S2. Knockdown of MKL1 promotes cell cycle progression at the G1-S phase transition. Additional file 3: Figure S3. MKL1 inhibits cell proliferation by altering the levels of cell cycle regulators. Additional file 4: Figure S4. MKL1 activates the transcriptional activity of p21 in a dose-dependent manner. Additional file 5: Figure S5. Knockdown of MKL1 results in upregulation of p21 expression.
