# Supplementary figures and images for: MKL1 inhibits cell cycle progression through p21 in podocytes
Source: BMC Mol Biol. 2015 Feb 12;16(1):1. doi: 10.1186/s12867-015-0029-5 (PMC4330937; doi:10.1186/s12867-015-0029-5)

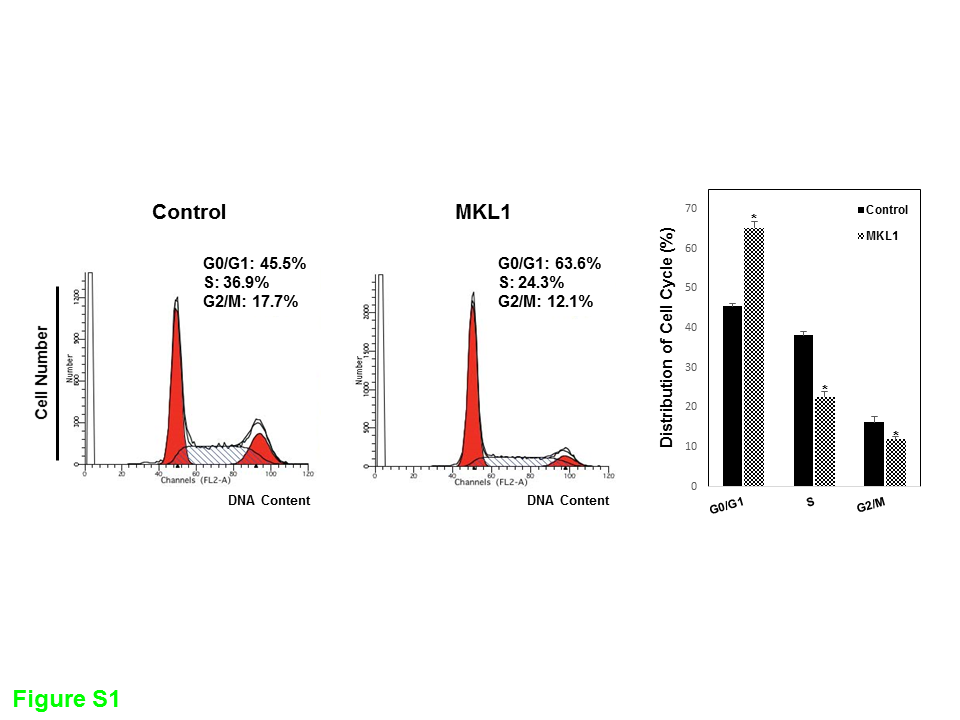

Supplement: Additional file 1: Figure S1. — Overexpression of MKL1 induces cell cycle delay at the G1-S phase transition. [file 12867_2015_29_MOESM1_ESM.tiff]

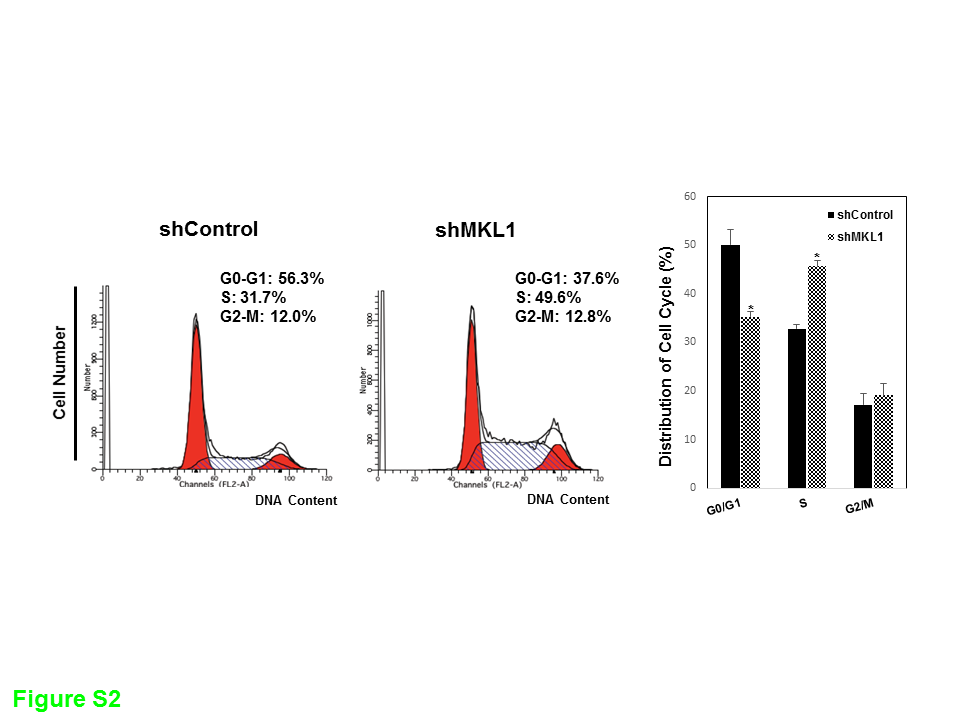

Supplement: Additional file 2: Figure S2. — Knockdown of MKL1 promotes cell cycle progression at the G1-S phase transition. [file 12867_2015_29_MOESM2_ESM.tiff]

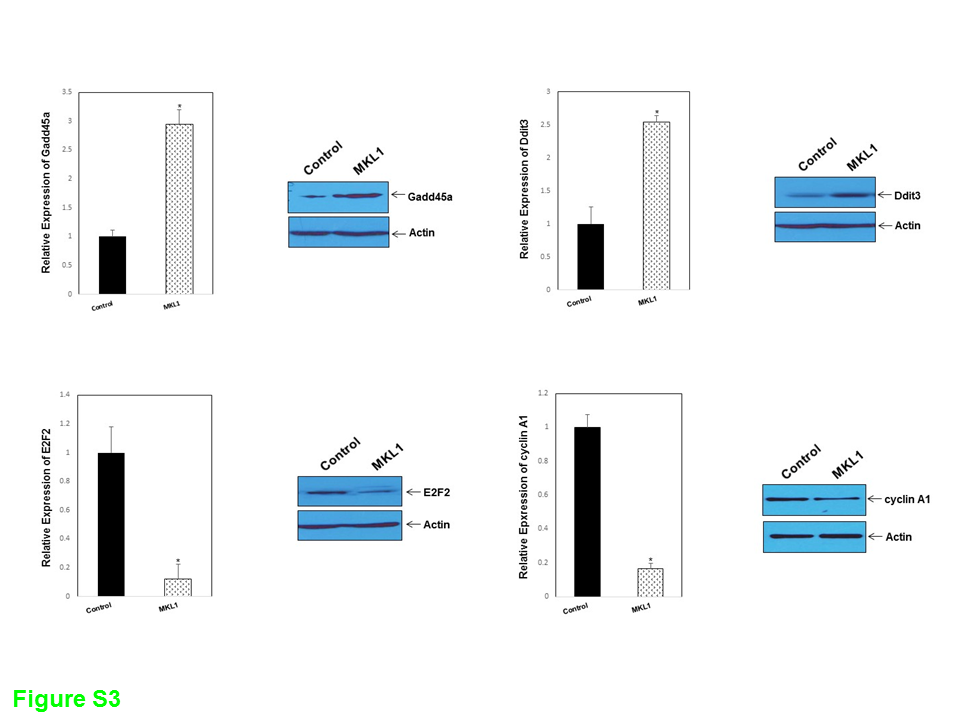

Supplement: Additional file 3: Figure S3. — MKL1 inhibits cell proliferation by altering the levels of cell cycle regulators. [file 12867_2015_29_MOESM3_ESM.tiff]

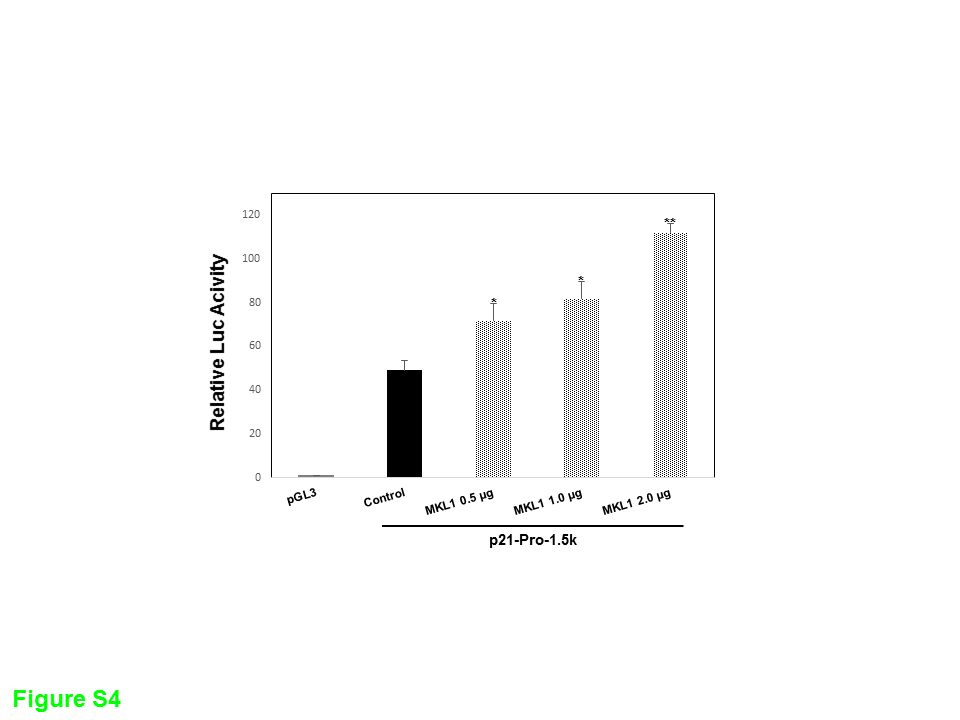

Supplement: Additional file 4: Figure S4. — MKL1 activates the transcriptional activity of p21 in a dose-dependent manner. [file 12867_2015_29_MOESM4_ESM.tiff]

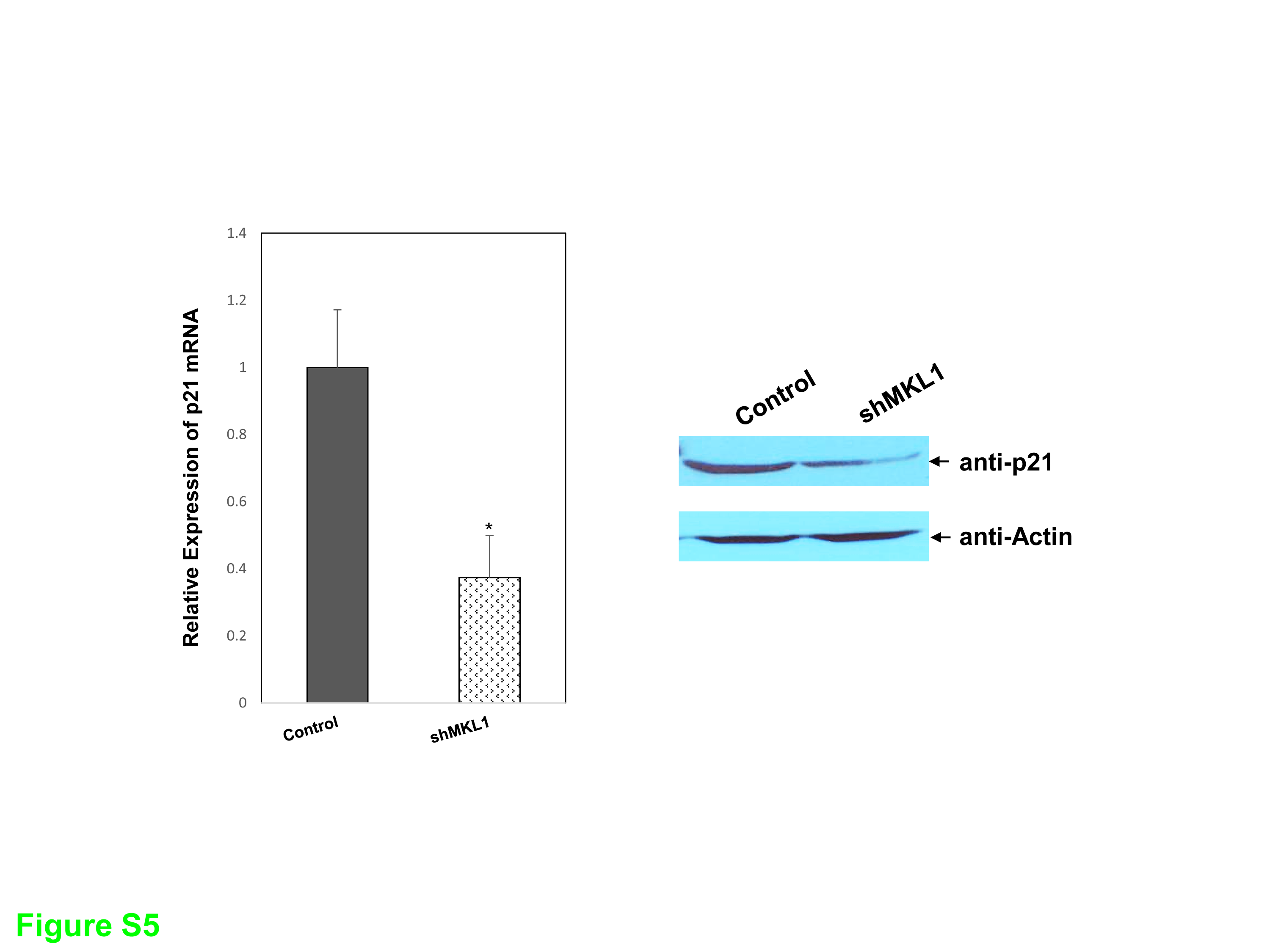

Supplement: Additional file 5: Figure S5. — Knockdown of MKL1 results in upregulation of p21 expression. [file 12867_2015_29_MOESM5_ESM.tiff]
